# *Dirofilaria* and *Wolbachia* in mosquitoes (Diptera: Culicidae) in central European Russia and on the Black Sea coast

**DOI:** 10.1051/parasite/2019002

**Published:** 2019-01-15

**Authors:** Elena Shaikevich, Anna Bogacheva, Ludmila Ganushkina

**Affiliations:** 1 Vavilov Institute of General Genetics, Russian Academy of Sciences Moscow 119991 Russia; 2 Moscow State University Moscow 119234 Russia; 3 Martsinovsky Institute of Medical Parasitology, Tropical and Vector-Borne Diseases, Sechenov First Moscow State Medical University Moscow 119435 Russia

**Keywords:** mosquitoes, *Dirofilaria repens*, *Dirofilaria immitis*, *Wolbachia pipientis*

## Abstract

Dirofilariasis is endemic in Russia, as well as in many other European countries. The aim of this study was to assess the ability of mosquitoes to transfer *Dirofilaria immitis* and *Dirofilaria repens* in regions with temperate and subtropical climates. The possible impact of the symbiotic bacterium *Wolbachia* on *Dirofilaria* transmission was also investigated. 5333 female mosquitoes were collected at 11 points in central European Russia and on the Black Sea coast during the period 2013–2017. Out of 20 mosquito species examined, 14 were infected with *D. repens* and 13 with *D. immitis*. Both species of *Dirofilaria* were found in different climatic regions. The total *Dirofilaria* spp. estimated infection rate (EIR) in the central part of Russia varied from 3.1% to 3.7% and, in the southern region, from 1.1% to 3.0%. The highest estimated infection rate was found in *Anopheles messeae*, the lowest in *Culex pipiens*. The greatest epidemiological danger was represented by *Aedes aegypti*, *Ae. geniculatus*, *An. messeae* and *Ae. communis*. Six out of 20 mosquito species were infected with *Wolbachia*. Pools of *Aedes albopictus*, *Cx. pipiens* and *Coquillettidia richiardii* were simultaneously infected with *Dirofilaria* and *Wolbachia*. After checking mosquitoes individually, it was found that there was no development of *Dirofilaria* to the infective larval stage in specimens infected with *Wolbachia*. Twenty-two *Dirofilaria*-infective pools were *Wolbachia*-free and only two mosquito pools were *Wolbachia*-infected. The potential for transmission of *Dirofilaria* in mosquito species naturally uninfected with the symbiotic bacterium *Wolbachia* is higher than in species infected with the bacterium.

## Abstract (fr)

La dirofilariose est endémique en Russie, ainsi que dans de nombreux autres pays européens. L’objectif de ce travail était d’étudier la capacité des moustiques à transférer *Dirofilaria immitis* et *Dirofilaria repens* dans les régions à climat tempéré et subtropical. L’impact possible de la bactérie symbiotique *Wolbachia* sur la transmission de *Dirofilaria* a également été étudié. 5333 moustiques femelles ont été collectés en 11 points en Russie centrale et sur la côte de la mer Noire au cours de la période 2013–2017. Sur les 20 espèces de moustiques examinées, 14 étaient infectées par *D. repens* et 13 par *D. immitis*. Les deux espèces de *Dirofilaria* ont été trouvées dans différentes régions climatiques. Le taux total d’infection estimé des *Dirofilaria* spp. dans la partie centrale de la Russie variait de 3,1 à 3,7 % et de 1,1 à 3,0 % dans le sud. Le taux d’infection estimé le plus élevé a été observé chez *Anopheles messeae* et le plus faible chez *Culex pipiens*. Le plus grand danger épidémiologique était représenté par *Aedes aegypti*, *Ae. geniculatus*, *An. messeae* et *Ae. communis*. Six espèces de moustiques sur 20 étaient infectées par *Wolbachia*. Des pools d’*Aedes albopictus*, *Cx. pipiens* et *Coquillettidia richiardii* étaient infectés simultanément par *Dirofilaria* et *Wolbachia*. Après avoir examiné les moustiques individuellement, il a été trouvé que les *Dirofilaria* ne se sont pas développés au stade larvaire infectant chez les spécimens infectés par *Wolbachia*. Vingt-deux pools infectés par *Dirofilaria* étaient indemnes de *Wolbachia* et seulement deux pools de moustiques étaient infectées par *Wolbachia*. Le potentiel de transmission de *Dirofilaria* chez les espèces de moustiques naturellement non infectées par la bactérie symbiotique *Wolbachia* est plus élevé que chez les espèces infectées par la bactérie.

## Introduction

Dirofilariasis is a vector-borne disease common in many countries on various continents [27, 42, 44, 60]. Sources of infection for mosquitoes are infected dogs, less often cats and wild canines (wolves, foxes, etc.). *Dirofilaria immitis* and *Dirofilaria repens* are transmitted by culicid mosquito species belonging to the *Culex*, *Aedes*, *Ochlerotatus*, *Anopheles*, *Coquillettidia*, *Armigeres* and *Psorophora* genera [42, 58, 69]. Vectors ingest microfilariae during a blood meal on an infected host. In mosquito Malpighian tubules, microfilariae develop to the third stage larvae (L3) [34]. The season for *Dirofilaria* transmission in the central part of Russia begins in late May to early June [26]. In order for the larvae to develop to L3, a sum of temperatures of 130 degrees-day [27] is necessary. L3 reach the salivary glands and proboscis from where they are transferred while feeding to another host [34, 43]. However, development of larvae to the infective stage does not always occur; *Dirofilaria* remain in the Malpighian tubules and do not undergo further development or are encapsulated by the immune system of mosquitoes, and may also die within a few hours of entering the intestine of a mosquito [18, 34]. Thus, only mosquitoes in which development has progressed to the third stage larvae (L3) can be considered epidemiologically competent vectors, and the larvae, infective.

*Dirofilaria* infection is endemic in Russia. Two species of *Dirofilaria* (*D. immitis* and *D. repens*) have been identified in humans [54, 63]. Prior to 2014, *D. repens* infection was detected in 850 people living permanently in 42 regions of the Russian Federation [54]. The first case of *D. immitis* was detected in 2015 in the Moscow region; an immature female was removed from a 14-month-old child [63]. The dirofilariasis zone in the north of the European part of Russia has advanced to 58° N [4, 10].

In Russia, mosquitoes infected with *Dirofilaria* have previously been investigated in the southern regions (Astrakhan, Rostov, Krasnodar Krai and Republic of Adygea) and the estimated infection rate (EIR) was 1.0%–14.0% [2, 24, 35]. Even though dirofilariasis is a concern in Russia, many areas have not been sufficiently studied. Also, there are no data on the species of mosquito that are potential vectors of dirofilarial worms. Identification of mosquitoes in all cases was conducted only to the genera level: *D. immitis* and *D. repens* have been detected in the *Culex*, *Aedes* and *Anopheles* genera and the EIR was established as 1.9%–7.0%, 2.3%–6.7%, and 0.6%–3.4%, respectively [2, 24, 35].

An endosymbiotic, maternally inherited bacterium, *Wolbachia pipientis* (Rickettsiales: Rickettsiaceae), hereafter *Wolbachia*, infects filarial nematodes and many insects, including some mosquito species. *Wolbachia* is required for the development and survival of filarial nematodes [61], whereas its symbiotic relationship with mosquitoes is largely parasitic [65]. Among the culicid mosquito species, *Culex pipiens, Cx. quinquefasciatus* and *Ae. albopictus* are known to be infected with *Wolbachia* [32, 67] and considered as vectors for *Dirofilaria* [13–15, 28, 45, 50, 69]. However, it was found that *Culex pipiens* f. *molestus* from Madeira, Portugal was unable to support the full development of *D. immitis*, both in nature and after experimental infection with *D. immitis* [29]. In continental Portugal, *Cx. pipiens* were found to be infected with *D. immitis*, but were not potentially infective; filarial DNA was detected only in the abdomen and not in thorax-head samples [25]. However, *D. immitis* microfilariae development to the L3 stage has recently been found in the thorax-head of one *Cx. pipiens* f. *pipiens* from Spain [9]. The hypothesis concerning the influence of *Wolbachia* on the transmission of *Dirofilaria* by *Cx. pipiens* mosquitoes in nature requires further confirmation, particularly in view of the limited number of infected specimens [9] and the absence of 100% *Wolbachia* infection of *Cx. pipiens* in nature [22, 56]. There are only three studies that have focused on investigating simultaneous infection with native *Wolbachia* and *Dirofilaria* in mosquitoes from natural populations [22, 23, 51]. Therefore, the effect of co-infection with native *Wolbachia* on mosquito vector competence for *Dirofilaria* remains unclear.

Prior to clarifying whether naturally occurring *Wolbachia* has any influence on filarial susceptibility or the development of *Dirofilaria* to the infective stage in the vectors, it is necessary to understand *Wolbachia*-mosquito interactions, which mosquito species are infected with the bacterium, the variability of bacterial strains, and the frequency with which *Wolbachia* occurs in mosquito populations.

The objectives of the current study were to examine mosquito fauna and to identify mosquito species that can potentially transmit filarial worms in rural and urban localities in the central part of European Russia compared with the Black Sea resorts, and to evaluate epidemiologically dangerous mosquito species in which larvae develop to the infective (L3) stage. All mosquito species were screened to determine their *Wolbachia* infection status.

## Materials and methods

### Mosquito sampling and taxa discrimination

**Figure 1.**
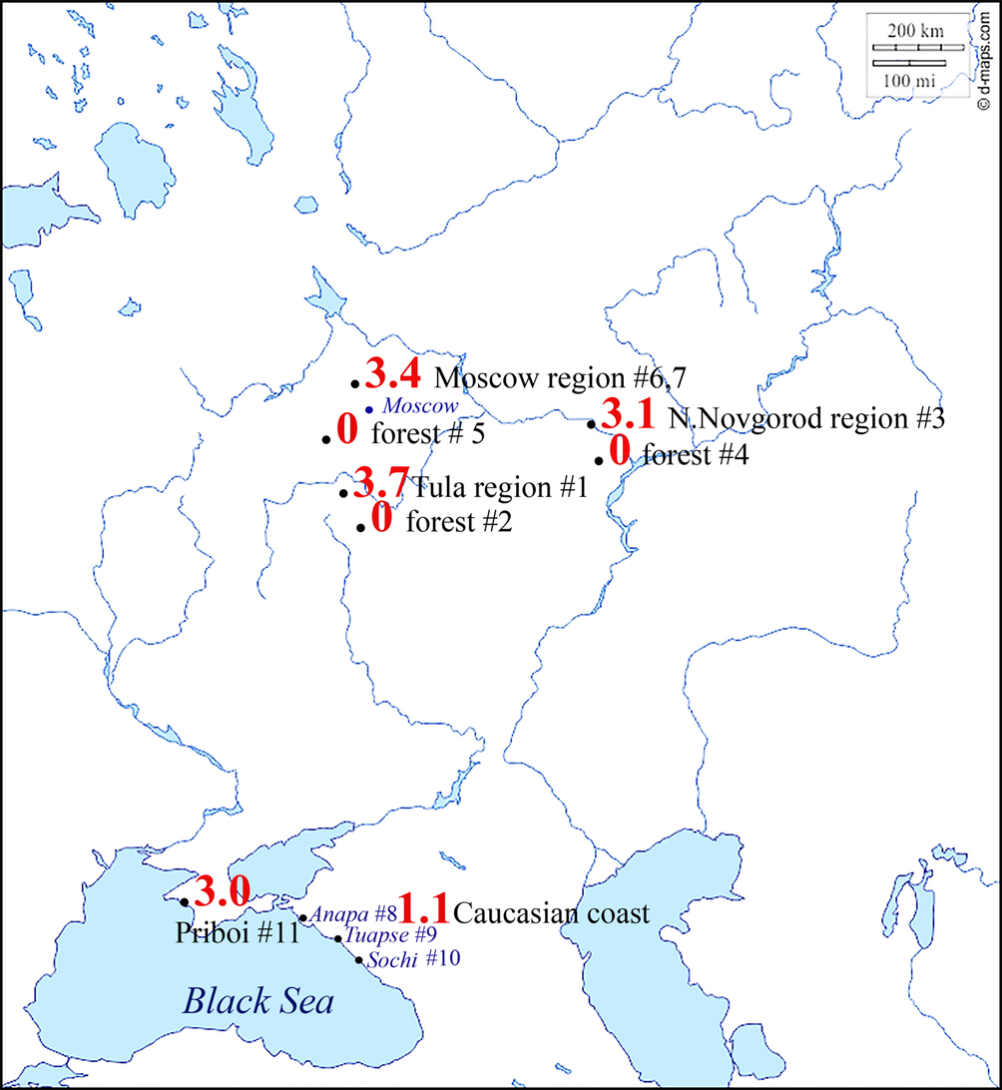
Map of mosquito sample sites and *Dirofilaria* infection rates (EIRs). EIR values for total *D. immitis* and *D. repens* are indicated in red. The exact names and geographical coordinates of the places of collection #1–11 are presented in SM1.

Mosquitoes were captured in the Tula region, Nizhniy Novgorod region, Moscow region and on the Black Sea coast (Fig. 1, SM1). The climate in the studied regions in the central part of the country is moderately continental with clear seasonality; the average temperature in July is +19 °C, and in January −10 °C. At the resorts of the Black Sea coast, the climate is mild Mediterranean and subtropical with average temperatures in July of +24 °C and in January +3 °C. Collection of mosquitoes in the central part of Russia was conducted throughout the warm season in 2013–2017, and in the southern part for one month at each point in 2012–2013 and 2016. Exact locations and months of gathering are presented in SM1.

Sampling locations in the Tula, Nizhniy Novgorod and Moscow regions were typical areas for a large number of dogs to be found (gardens of private houses, forests and parks) and natural forests as far as 6–8 km from rural and urban areas. At one of the sampling points in the Moscow region (#5 Fig. 1), there was a kennel for stray dogs; this was located in the immediate vicinity of the forest where mosquito collections took place. To compare the infection rate of mosquitoes in urban and rural areas, we collected mosquitoes near human habitations and in forests.

Mosquito collection sites in the south were located in human settlements in a resort area. At the recreation centre “Priboi” (#11 Fig. 1), lakes and ponds are located at a distance of 200–500 m from the collection site and flying mosquito imagoes were observed here. On the Black Sea coast of the Caucasus (#8, 9, 10 Fig. 1) in Anapa, Tuapse and in Sochi, mosquitoes were collected in both urban and rural areas.

At all collection sites, the mosquitoes were captured using a suck tube by human landing during the most active attacking period from 6 pm to 9 pm several times during each month. After trapping, the mosquitoes were frozen at −19 °C for 20–30 min and, afterwards, were identified using taxonomic keys [31]. The specific name of the tribe Aedini is presented according to the studies of Wilkerson et al. [66]. Identification of the *molestus* and *pipiens* forms of *Cx. pipiens* and *Cx. torrentium* was conducted genetically using a PCR-RFLP assay, based on the DNA variability of the *COI* gene, as described previously [55, 56]. Representatives of *Anopheles maculipennis* complex were identified using an ITS2 PCR-RFLP [47].

### Molecular *Dirofilaria* spp. screening

The collected mosquitoes were grouped according to species, collection site and year; there were up to six specimens/pool, usually five. The thorax-head and abdomen of each mosquito in the group were dissected and formed the pool. In some cases, individual thorax-heads were analysed. DNA extraction was performed using the DIAtom™ DNA Prep kit (Isogen, Russia). Extraction was conducted separately for the abdomens and thorax-heads in order to determine infected and infective mosquito specimens, respectively. For the PCR analysis, we used 5333 female mosquitoes which were divided into 1095 pools. Each pool was tested separately to identify *D. immitis* and *D. repens* using the following primers: DIR-3: F-5′–CCGGTAGACCATGGCATTAT–3′ and DIR-4: R-5′–CGGTCTTGGACGTTTGGTTA–3′ for the *D. repens* DNA repeat region [64] and COIintF – 5′–TGATTGGTGGTTTTGGTAA–3′ and COIintR – 5′–ATAAGTACGAGTATCAATATC–3′ for detection of the *COI* gene in the mtDNA of *D. immitis* [46]. The PCR was run on a GeneAmpR PCR System 2700 thermal cycler (Applied Biosystems, USA) with a GenPak PCR MasterMix Core PCR kit (Isogen, Russia) for amplification, according to the manufacturer’s instructions. PCR was performed in 25-μl reaction mixtures, containing 1.5 mM of MgCl_2_, 10 pmol of each primer and 20–50 ng of mosquito genomic DNA. PCR protocols were as follows: primary denaturing at 94 °C for 5 min and then 48 cycles of 94 °C for 30 s, annealing at 50 °C for 30 s and extension at 72 °C for 60 s, and a final extension at 72 °C for 5 min for *D. repens*; primary denaturing at 94 °C for 5 min and then 30 cycles of 94 °C for 1 min, 50 °C for 2 min and 72 °C for 3 min, and final extension for 5 min at 72 °C for *D. immitis.* Negative and positive controls were used in each PCR analysis to avoid false-positive results. The positive control used in the study was obtained from adult *D. repens* isolated from a dog. The presence of filarial DNA was confirmed using 1.5% agarose gel electrophoresis. Resulting amplicon sizes were 656 bp for *D. immitis* and 246 bp for *D. repens*.

### Calculation of the infection rates

Minimum infection rates (MIRs) were calculated using the following standard formula: (number of positive mosquito pools)/(total number of mosquitoes tested) × 100 [13]. Estimated infection rates (EIRs) were calculated using the following formula: 1 − (1 − *x/m)* 1/*k* [20], where *x* is the number of positive pools, *m* is the number of pools tested, and *k* is the pool size. In the text, all EIRs are given per 100 specimens. Corresponding 95% confidence intervals (95% CIs) were calculated by the modified Wald method, using GraphPad Scientific software. EIR values with corresponding 95% CIs were calculated for all analysed pools per mosquito and *Dirofilaria* species. Host effectiveness was determined as the number of infectious mosquito pools with L3 larvae as a proportion of the total number of mosquitoes studied × 100.

### Molecular *Wolbachia* screening

*Wolbachia* infection was detected in a sub-sample of 2926 individuals (633 pools) of 20 mosquito species by PCR with an Encyclo PCR kit (Evrogen, Russia), using the *wsp*-specific primers wsp-81F and wsp-691R [8]. In cases where *Dirofilaria* DNA was detected in pooled abdomens from the mosquitoes, pools of abdomen and individual thorax-heads were tested for *Wolbachia* infection. In other cases, pooled mosquito thorax-heads were analysed. The PCR fragments were purified from agarose gel with a Clean-Up Extraction Kit (Evrogen, Russia) and were sequenced using the BigDye Termination kit 3.1 (Applied Biosystems, USA) in order to distinguish *Wolbachia* of mosquito and of filarial nematode origins. Sequences of the *Wolbachia wsp* locus were deposited in GenBank under numbers MF989984–MF989989.

To distinguish between two strains of *Wolbachia* in *Ae. albopictus*, multi-primer PCR was used [70]; primers 383F and 183F were paired with wsp-691R to allow the separation of *w*AlbA and *w*AlbB *Wolbachia* strains from *Ae. albopictus*. For the *w*AlbA strain, a fragment size of 379 bp was found and, for the *w*AlbB strain, an amplicon length of 501 bp was found.

The *w*Pip infections in *Cx. pipiens* were genotyped in a subsample of 24 individuals representative of *Dirofilaria*-positive pools and assigned to the *w*Pip-II and *w*Pip-IV groups, using PCR-RFLP assays based on two *w*Pip markers, *ank2* and *pk1,* as previously described [3, 56].

## Results

### *Dirofilaria* spp. infection in mosquitoes

The collected mosquitoes included 20 species; 16 species were in the central part of Russia and seven species on the Black Sea coast. The most abundant mosquito species in the temperate climate region was determined to be *Ae. cantans*. In the subtropical climate on the Black Sea coast, the most abundant sampled mosquito species was *Ae. albopictus*, followed by *Cx. pipiens* and *Cx. modestus* (Table 1). Filarial DNA was found in 15 species belonging to four genera with the total EIR for both *Dirofilaria* infections calculated as 2.71 (95% CI, 2.18–3.03) (Table 2). The highest EIR values occurred with the species *An. messeae, Ae. aegypti, Ae. geniculatus* and *Ae. cataphylla*, and were estimated as 8.67, 5.33, 4.85 and 4.12, respectively (Table 2). *D. repens* infected 14 of 15 mosquito species (EIR = 1.17, 61 positive pools) and *D. immitis* 13 species (EIR = 1.47, 76 positive pools). Both *Dirofilaria* species were found in one pool of *Ae. cantans* (twice), *Ae. geniculatus* (once), *Cx. pipiens* (once), *Ae. intrudens* (once) and *Cq. richiardii* (once). More than one abdomen pool positive with *D. repens* was detected from *Ae. cantans*, *Ae. cataphylla*, *Ae. intrudens* and *Ae. geniculatus*; with *D. immitis* from *Ae. cantans*, *Ae. intrudens*, *Ae. communis*, *Ae. albopictus*, *Cx. pipiens*, *Ae. geniculatus*, *Cq. richiardii*, *Cx. modestus* and *Ae. cinereus* (Table 2).

**Table 1.** Mosquito species composition and their collected numbers in studied regions.

No.	Mosquito species	Tula region	N. Novgorod region	Moscow region	Total in Central European Russia (%)	Black Sea coast Caucasus	Crimean peninsula, Priboi	Total in southern regions (%)
1	*Anopheles messeae* (Falleroni)	33	23	6	62 (1.44)	5	0	5 (0.48)
2	*Coquillettidia richiardii* (Ficalbi)	57	87	3	147 (3.42)	25	12	37 (3.57)
3	*Aedes* (*Stegomyia*) *albopictus* (Scuse)	0	0	0	0	366	0	366 (35.36)
4	*Aedes* (*Stegomyia*) *aegypti* (Linnaeus)	0	0	0	0	21	0	21 (2.03)
5	*Aedes (Aedes*) *cinereus* (Meigen)	144	93	22	259 (6.03)	0	0	0
6	*Aedes* (*Aedimorphus*) *vexans* (Meigen)	125	46	8	179 (4.16)	0	0	0
7	*Aedes* (*Finlaya*) *geniculatus* (Olivier)	200	3	0	203 (4.72)	0	0	0
8	*Aedes* (*Ochlerotatus*) *cantans* (Meigen)	1140	337	299	1776 (41.32)	0	0	0
9	*Aedes* (*Ochlerotatus*) *communis* (de Geer)	88	67	152	307 (7.14)	0	0	0
10	*Aedes* (*Ochlerotatus*) *punctor* (Kirby)	50	0	0	50 (1.16)	0	0	0
11	*Aedes* (*Ochlerotatus*) *intrudens* (Dyar)	156	282	44	482 (11.21)	0	0	0
12	*Aedes* (*Ochlerotatus*) *cataphylla* (Dyar)	218	13	5	236 (5.49)	0	0	0
13	*Aedes* (*Ochlerotatus*) *leucomelas* (Meigen)	62	0	0	62 (1.44)	0	0	0
14	*Aedes* (*Ochlerotatus*) *excrucians* (Walker)	34	7	27	68 (1.58)	0	0	0
15	*Aedes* (*Ochlerotatus*) *caspius* (Pallas)	0	0	0	0	140	6	146 (14.11)
16	*Aedes* (*Ochlerotatus*) *diantaeus* (Howard, Dyar & Knab)	0	117	11	128 (2.98)	0	0	0
17	*Aedes* (*Ochlerotatus*) *sticticus* (Meigen)	54	0	3	57 (1.33)	0	0	0
18	*Culex* (*Culex) pipiens* (Linnaeus)	270	9	0	279 (6.49)	70	167	237 (22.89)
19	*Culex* (*Culex) torrentium* (Martini)	3	0	0	3 (0.07)	0	0	0
20	*Culex* (*Barraudius*) *modestus* (Ficalbi)	0	0	0	0	0	223	223 (21.55)
	**Total**	2634	1084	580	4298 (100)	627	408	1035 (100)

**Table 2.** Mosquito species and infection with *D. immitis* and *D. repens*.

Mosquito species	Number of indiv. mosquitoes	Number of pools	Average number of specimens per pool	Pools positive for *D. repens*			Pools positive for *D. immitis*			Total infection with either *D. immitis* and *D. repens*		
				Number of abdomen pools	Number of head-thorax pools	EIR (95% CI)	Number of abdomen pools	Number of head-thorax pools	EIR (95% CI)	MIR (%)	EIR (95% CI)	Host effectiveness** (%)
*An. messeae*	67	15	4.47	1	1	3.15 (0.21–10.86)	3	0	4.87 (1.03–12.87)	7.46	8.67 (2.86–16.68)	1.49
*Ae. aegypti*	21	4	5.25	0	1	5.33 (0.01–24.42)	0	0	0	4.76	5.33 (<0.01–24.42)	4.76
*Ae. geniculatus*	203	43	4.72	2	2	2.05 (0.59–5.13)	2	3	2.59 (0.9–5.8)	4.43	4.85 (2.23–8.33)	2.46
*Ae. cataphylla*	236	49	4.82	6	0	2.67 (1.04–5.56)	3	0	1.3 (0.26–3.85)	3.81	4.12 (1.91–7.19)	0
*Cq. richiardii*	184	40	4.6	2	0	1.11 (0.04–4.13)	3	1	2.27 (0.65–5.65)	3.26	3.47 (1.34–7.09)	0.54
*Cx. modestus*	223	45	4.96	3	0	1.38 (0.27–4.06)	2	2	1.86 (0.54–4.68)	3.14	3,35 (1.40–6.46)	0.89
*Ae. cantans*	1776	356	4.99	25(8*)	11(8*)	1.63 (1.08–2.28)	21(1*)	4(1*)	1.9 (0.9–2.01)	2.93	3.11 (2.23–3.83)	0.84
*Ae. communis*	307	64	4.79	3(1*)	1*	0.99 (0.2–2.97)	3	3	2.03 (0.8–4.3)	2.93	3.11 (1.47–5.56)	1.3
*Ae. intrudens*	482	98	4.92	4	1	1.06 (0.37–2.48)	8	1	1.94 (0.93–3.57)	2.9	3.08(1.69–4.86)	0.41
*Ae. vexans*	179	37	4.84	2	0	1.14 (0.04–4.24)	3(1*)	1*	1.73 (0.35–5,04)	2.79	2.96 (1.02–6.55)	0.56
*Ae. cinereus*	259	54	4.79	1	0	0.39 (<0.01–2.38)	4	0	1,59 (0.46–4.05)	1.93	2.01 (0.70–4.57)	0
*Ae. albopictus*	366	74	4.95	0	1	0.27 (<0.01–1.69)	5	0	1.4 (0.49–3.25)	1.64	1.69 (0.67–3.62)	0.27
*Ae. leucomelas*	62	13	4.77	0	0	0	1	0	1.66 (<0.01–9.41)	1.61	1.66 (<0.01–9.41)	0
*Ae. excrucians*	68	15	4.53	1	0	1.51 (<0.01–8.63)	0	0	0	1.47	1.51 (<0.01–8.63)	0
*Cx. pipiens*	516	104	4.96	1	1	0.39 (0.01–1.5)	5	0	0.99 (0.35–2.32)	1.36	1.39 (0.60–2.83)	0.19
*Ae. sticticus*	57	13	4.38	0	0	0	0	0	0	0	0	0
*Ae. caspius*	146	30	4.87	0	0	0	0	0	0	0	0	0
*Ae. punctor*	50	11	4.55	0	0	0	0	0	0	0	0	0
*Ae. diantaeus*	128	27	4.74	0	0	0	0	0	0	0	0	0
*Cx. torrentium*	3	3	1	0	0	0	0	0	0	0	0	0
Total	5333	1095	4.87	51(9*)	19(9*)	1.17 (0.89–1.47)	63(2*)	15(2*)	1.47 (1.14–1.78)	2.57	2.71 (2.18–3.03)	0.64
						MIR = 1.14			MIR = 1.43			

* Inclusive pools, in which infection was detected in both abdomens and head-thorax pools;

** Host effectiveness – proportion of infectious mosquitoes with L3 larvae in total number of studied mosquitoes (%).

If we compare infection in the abdomen with the thorax-head, *D. immitis* DNA was detected in 5.75% of tested abdomen pools and 1.37% of tested thorax-head pools. *D. repens* was found in 4.66% of tested abdomen pools and in 1.74% of tested thorax-head pools. The species *Ae. cantans*, *Ae. geniculatus*, *Ae. communis* and *Cx. modestus* had more than one positive thorax-head pool (15, 5, 4 and 2 pools, respectively). *Ae. cantans*, *Ae. communis* and *Ae. vexans* had *Dirofilaria* in the abdomen and in the thorax-head in the same pools. In four species, *Ae. cataphylla*, *Ae. leucomelas*, *Ae. excrucians* and *Ae. cinereus*, microfilariae were only found in the abdomen, with EIR values of 1.51–4.12 (Table 2).

The EIR results for *Dirofilaria* in mosquitoes from specific collection sites are presented in an additional file (SM2). In the Nizhny Novgorod region (Fig. 1 #3), EIRs for *D. repens* and *D. immitis* were 1.23 and 1.92, respectively. In the Tula region (Fig. 1 #1), EIRs for *D. repens* and *D. immitis* were 1.78 and 1.82, respectively. In the Moscow region (Fig. 1 #6, 7), the EIR for *D. repens* was 1.63 and for *D. immitis* 1.97, in a dog kennel in the forest near Moscow (Fig. 1 #5) infected mosquitoes were not found. The first infected mosquitoes were recorded in May and the last in August–September. In the forest zones of the Nizhny Novgorod and Tula regions, with a sample of 525 specimens, no infected mosquitoes were found (SM2). At the resorts of the Caucasian Black Sea coast, in Anapa, Tuapse and Sochi (Fig. 1 #8, 9, 10), the rates of mosquito infection were much lower; EIR values were 0.32 for *D. repens* and 0.81 for *D. immitis*. However, at the recreation center “Priboi” (Fig. 1 #11), *D. repens* had an EIR of 0.99 and *D. immitis* 2.04; this is comparable to values in the central regions of Russia (Fig. 1).

### *Wolbachia* infection in mosquitoes

The presence of *Wolbachia* was found in six out of 20 studied mosquito species. 93% of all tested *Cx. pipiens* were infected with *Wolbachia*, followed by *Cq. richiardii* (68%), *Ae. albopictus* (56%), *Ae. cinereus* (37%), *Cx. modestus* (7%) and *Ae. cantans* (3%). Specific sample sites and screening results are presented in an additional file (SM3). Sequences of *Wolbachia wsp* genes from all six mosquito species demonstrated that all bacteria belonged to supergroups A or B, which were shared between arthropods (Table 3). No filarial bacteria were amplified. Two *Wolbachia* strains were present in studied *Ae. albopictus* (*w*AlbA and *w*AlbB), *w*Pip-II in *Cx. pipiens* f. *pipiens* and *w*Pip-IV in *Cx. pipiens* f. *molestus.* Based on the *wsp* gene sequence, *Wolbachia* strains in *Cq. richiardii, Ae. cinereus* and *Ae. cantans* differ from *w*Alb and *w*Pip, so we named these *w*Crich, *w*Acin and *w*Ocan, respectively (pubmlst.org/wolbachia).

**Table 3.** Positive pools for both *Dirofilaria* and *Wolbachia*. Bold, samples positive for *Wolbachia*.

Species	Pools positive for *D. repens* (inclusive heads)	Pools positive for *D. immitis* (inclusive heads)	Pools positive for *Wolbachia*	*Wolbachia* supergroup
*Ae. cinereus*	0	1	0	
*Ae. vexans*	1	0	0	
*Ae. geniculatus*	4 (2)	5 (2)	0	
*Ae. cantans*	26 (9)	9 (3)	0	
*Ae. cataphila*	1	0	0	
*Ae. intrudens*	2	6	0	
*Ae. communis*	3 (1)	1	0	
*Ae. excrucians*	1	0	0	
*Ae. aegypti*	(1)	0	0	
***Ae. albopictus***	**(1)**	**5**	**6**	**A and B**
*An. messeae*	2 (1)	2	0	
***Cq. richiardii***	**1**	**4 (1)**	**2**	**B**
***Cx. pipiens*** s.l.	**2(1)**	**4**	**7**	**B**
*Cx. modestus*	3	4 (2)	0	
Total	48 (16)	41 (8)	15	

A total of 90 *Dirofilaria* positive abdomen and thorax-heads pools were analyzed for simultaneous infection with *Wolbachia* (Table 3). Seventy five of the *Dirofilaria* positive pools (83%), including *Ae. cinereus, Ae. cantans* and *Cx. modestus*, were free from *Wolbachia*. Fifteen pools of *Ae. albopictus, Cx. pipiens* and *Cq. richiardii* (17%) were positive for both *Wolbachia* and at least one *Dirofilaria* species. *Dirofilaria* was found in 22 thorax-head pools of mosquitoes uninfected with *Wolbachia* and in two thorax-head pools which were positive for *Wolbachia*.

In order to investigate a possible association between the occurrence of *Wolbachia* and the development of *Dirofilaria* to the infective third larval stage (L3) within mosquitoes, we tested the thorax-heads of individual specimens from 12 pools: 25 individuals (five pools) of *Ae. albopictus,* 23 individuals (five pools**)** of *Cx. pipiens* (21 f. *pipiens* and two f. *molestus*), and 11 individuals (two pools) of *Cq. richiardii* (SM 4). There was no possibility to study one pool of *Ae. albopictus* (collected in Sochi 2012) infected with *D. repens* and two pools of *Cx. pipiens* (collected in Tula 2014) – one infected with *D. repens* and one infected with *D. immitis* individually. The development of *D. immitis* to the infective stage (L3) was successful only in one thorax-head of *Wolbachia*-free *Cq. richiardii* (No. 11’-1, SM4), although a pool of five mosquito abdomens gave a positive signal for both *D. repens* and *D. immitis*. Neither *D. immitis* nor *D. repens* were found in other individual thorax-heads; parasites were found only in pooled abdomens.

## Discussion

### Mosquito species and *Dirofilaria* infection

The detection of infection with *D. repens* and *D. immitis* was tested in 5333 mosquitoes comprising 1095 pools and representing 20 species collected in geographically remote locations in a temperate and sub-tropical climate. This is the first large-scale study of the infection of mosquitoes in the European part of Russia involving identification of the mosquito species. The published results on mosquito infestation in Europe, including Turkey, in comparison with our data are presented in Table 4. Previously, *Ae. cataphylla*, *Ae. cinereus*, *Ae. excrucians*, *Ae. leucomelas*, *Ae. punctor* and *Ae. diantaeus* mosquitoes were studied in Europe for the presence of *Dirofilaria*, but no positive samples were detected. In our study, infection with *Dirofilaria* was newly detected in the first four of these mosquito species. However, development of the larvae did not reach the infectious stage.

**Table 4.** Published results about *Dirofilaria* in mosquito species in Europe, including Turkey, in comparison with data obtained in this study.

Species	N indiv./pools	*D. repens*	*D. immitis*	Host effectiveness	Country, references
*Cx. pipiens*	516/104	EIR = 0.39	EIR = 0.99	0.19	This study
*Cx. pipiens*	1108/412	MIR = 0.27	MIR = 0.27	0.27*	Italy 2002–2003 [12]
*Cx. pipiens* (s.l.)/*torrentium*	2663/132	EIR = 0.88	EIR = 0.47		Moldova 2010–2016 [58]
*Cx. pipiens*	1595/1123		EIR = 0.50		Continental Portugal 2011–2013 [25]
*Cx. pipiens*	2589		MIR = 0.12	0.12*	Turkey 2008–2009 [69]
*Cx. pipiens*	37,865/835	MIR = 0.01*	MIR = 0.04*		Italy 2010 [38]
*Cx. pipiens*	5568/115	MIR = 0.02*	MIR = 0.18*		Serbia 2013 [37]
*Cx. pipiens complex*	2539/187	MIR = 0.28*			Slovakia 2015–2017 [11]
*Cx. pipiens/Cx. torrentium*	12,292/554		MIR = 0.02*		Germany 2011–2013 [36]
*Cx. pipiens*	136/11		EIR = 0.58		Belarus 2015 [59]
*Cx. pipiens*	666		MIR = 0.3*		Spain 2004–2006 [46]
*Cx. pipiens*	604		MIR = 0.17*	0.17*	Spain 2012–2013 [9]
*An. messeae*	67/15	EIR = 3.15	EIR = 4.87	1.49	This study
*An. maculipennis* s.l.	400/114		EIR = 3.12	1.25*	Continental Portugal 2011–2013 [25]
*An. maculipennis*	136/28	MIR = 1.47*			Slovakia 2015–2017 [11]
*An. maculipennis* s.l.	947/ 62	EIR = 4.91	EIR = 2.01		Moldova 2010–2016 [58]
*Ae. vexans*	179/37	EIR = 1.14	EIR = 1.73	0.56	This study
*Ae. vexans*	3179		MIR = 0.41	0.35*	Turkey 2008–2009 [70]
*Ae. vexans*	720/25		MIR = 0.14*		Italy 2010 [38]
*Ae. vexans*	405/19	MIR = 0.25*			Serbia 2013 [37]
*Ae. vexans*	12,042	MIR = 0.03			Czech Republic 2009–2011 [52]
*Ae. vexans*	314/ 33	EIR = 1.68			Moldova 2010–2016 [58]
*Ae. vexans*	1750/35	MIR = 0.06			Slovakia 2012 [7]
*Ae. vexans*	96/20		MIR = 1.04		Turkey 2008 [6]
*Ae. caspius*	146/30	EIR = 0	EIR = 0	0	This study
*Ae. caspius*	26/13	EIR = 22.64			Moldova 2010–2016 [58]
*Ae. caspius*	270/193		EIR = 3.73	1.48*	Continental Portugal 2011–2013 [25]
*Ae. caspius*	2264/92		MIR = 0.18*		Italy 2010 [38]
*Ae. caspius*	195/13		MIR = 0.5*		Serbia 2013 [37]
*Cq. richiardii*	184/40	EIR = 1.11	EIR = 2.27	0.54	This study
*Cq. richiardii*	34/7		MIR = 2.94*		Serbia 2013 [37]
*Cq. richiardii*	48/26		MIR = 2.08*		Slovakia 2015–2017 [11]
*Cq. richiardii*	19/11	EIR = 16.25			Moldova 2010–2016 [58]
*Ae. cantans*	1776/356	EIR = 1.63	EIR = 1.39	0.84	This study
*Ae. cantans*	15/5	EIR = 14.84			Moldova 2010–2016 [58]
*Ae. sticticus*	57/13	EIR = 0	EIR = 0	0	This study
*Ae. sticticus*	24/7	EIR = 4.43			Moldova 2010–2016 [58]
*Ae. sticticus*	120/7	MIR = 0.83*			Serbia 2013 [37]
*Ae. sticticus*	414/41	MIR = 0.24*	MIR = 0.24*		Slovakia 2015–2017 [11]
*Cx. modestus*	223/45	EIR = 1.38	EIR = 1.86	0.89	This study
*Cx. modestus*	203/25	EIR = 3.26			Moldova 2010–2016 [58]
*Ae. geniculatus*	203/43	EIR = 2.05	EIR = 2.59	2.46	This study
*Ae. geniculatus*	26/10	EIR = 7.45			Moldova 2010–2016 [58]
*Ae. albopictus*	366/74	EIR = 0.27	EIR = 1.4	0.27	This study
*Ae. albopictus*	2534/336	0	MIR = 3.19*	0.87*	Italy 2000–2002 [12, 13]
*Ae. albopictus*	436/436	MIR = 0.92*	MIR = 0.69*	1.15*	Italy 2002–2003 [16]
*Ae. albopictus*	528/98		MIR = 0.19*		Italy 2005 [41]
*Ae. albopictus*	175/35		MIR = 1.14	0.51*	Italy 2011 [28]

* Number calculated based on the results published by the authors.

*Ae. intrudens* and *Ae. communis* were firstly studied here as vectors of *Dirofilaria*; their EIR values were 3.08 and 3.11, respectively. It should be noted that their epidemiological significance is confirmed by the presence of third-stage larvae in the thorax-heads. *Ae. intrudens* and *Ae. communis* host effectiveness was 0.41 and 1.3, respectively.

Special attention should be paid to *Ae. aegypti* mosquitoes, which were found in Russia on the Black Sea coast of the Caucasus in 2000 [53]. As far as we know, the infection of natural populations of *Ae. aegypti* has not been studied. Based on a small sample (21 females), the EIR of this species of mosquitoes was reported as 5.33.

In our study, the most abundant species of mosquito was *Ae. cantans* (1776 specimens out of 5333) with an infection rate that was not high; the EIR for *D. repens* was 1.63 and for *D. immitis* 1.39. Infection of *Cq. richiardii* mosquitoes with *D. immitis* (EIR = 2.27) is comparable to data from Serbia [37] and Slovakia [11]. However, in Moldova [58], mosquitoes of these three species were infected with *Dirofilaria* to a greater extent than in our results for Russia (Table 4). In our study, *Ae. strictus* mosquitoes were not infected, in contrast with data from Serbia, Slovakia and especially from Moldova. The epidemiological significance of *Ae. vexans*, widely distributed in Europe, as a vector of *Dirofilaria* was minor. Its infection rate in different countries varied from 0.03 to 1.68 (Table 4). In Russia, in the Nizhny Novgorod region, there were no infected mosquitoes of this species; in Tula, *D. repens* was found with EIR of 0.81 and *D. immitis* with EIR 2.52. In the Moscow region, of a small sample of three *Ae. vexans* females, one was infected with *D. repens*. The infection rate with *D. repens* in *Cx. modestus* and *Ae. geniculatus* was similar to infection of these mosquito species in Moldova [58]. The difference is that in our samples, both mosquito species were infected, not only with *D. repens*, but also with *D. immitis*.

In our study, the highest EIR value was found in *An. messeae.* Importantly, some *An. messeae* mosquitoes were collected in early May. The females that flew out after the winter diapause actively attacked both humans and dogs. In the Nizhny Novgorod region, 2 out of 12 females caught in May were infected with microfilariae. Somewhat different infection rates of this mosquito species were reported in Moldova (Table 4).

The lowest infection in our study was found in *Ae. albopictus* and *Cx. pipiens*. According to our data, *Ae. albopictus* mosquitoes were infected with *D. repens* with an EIR of 0.27 and, with *D. immitis*, an EIR of 1.4. In Italy, *Ae. albopictus* infected with *D. repens* [13], with *D. immitis* [28, 41] and with both species [16] were found with MIR values ranging from 0.69 to 3.19 (Table 4). According to our results, *Cx. pipiens* mosquitoes were infected with *D. repens* with EIR 0.39 and with *D. immitis* EIR 0.99. A comparable frequency was recorded in Italy, Turkey, Portugal, Germany, Moldova and the Republic of Belarus (Table 4).

Differences in infection rates in the same mosquito species from different regions could be connected with ecological factors, such as season, climate and geographical features, which are specific for each region [27], but also, perhaps to an even greater extent as shown by our results with *Ae. aegypti*, *Ae. cantans* and *Ae. vexans*, connected with the sample size.

### Infection in specific collection regions

When comparing the total infection of mosquitoes with *D. repens* and *D. immitis* by region (SM2), almost identical EIR results in the settlements of the Central region were noted, with some prevalence of mosquitoes infected with *D. immitis*. On the Black Sea coast of the Caucasus (Fig. 1 #8, 9, 10), mosquitoes were collected in a resort area where the number of dogs near our sample sites was negligible and the infection of mosquitoes was lower. Temperature is an important factor for the maintenance of dirofilariasis foci. However, the presence of definitive hosts (mainly domestic, office and stray dogs) basically determines one or another level of mosquito infection with *Dirofilaria*. This is confirmed by the absence of infection in mosquitoes collected in the forest at a distance of 8–10 km from settlements in the Nizhny Novgorod and Tula regions (Fig. 1, “forests” #2, 4). However, in the settlements (Fig. 1 #1, 3, 6, 7), mosquito infection was high >3%. Possible reasons for this are that circulation of the pathogen in the two woodlands does not occur, or wild canines are not affected by *Dirofilaria* or are affected to such a small extent that we could not discern infectionby examining the mosquito vectors.

In the Moscow region, one point was studied in the immediate vicinity of the dog kennel (Fig. 1 #5), located in a woodland 2 km away from the nearest settlement. Infected mosquitoes were not found. In this kennel, the dogs were treated for different infections, including *Dirofilaria*, and the infection from wild animals did not occur or was extremely low.

### Host effectiveness

According to our findings, under similar conditions (temperature and the presence of definitive hosts), the effectiveness of mosquitoes as vectors of *Dirofilaria* was not the same. There were five species of mosquitoes, *Ae. punctor, Ae. diantaeus, Ae. sticticus, Ae. caspius* and *Cx torrentium,* in which no infected samples were found. Absence of infection in *Ae sticticus* and *Ae. punctor* was probably associated with a small sample size (57 and 50 mosquitoes, respectively). *Cx. torrentium* rarely attack people, and with our collection method, the sample size was only three mosquitoes. However, of particular interest is the reason for the absence of infection in *Ae. diantaeus* and *Ae. caspius*, which were collected in sufficient numbers (117 and 146 mosquitoes) and not in the natural forests. Another interesting finding was the absence of infection in *Ae. diantaeus*, which were mainly collected in the Nizhny Novgorod region, where other mosquito species of the same biotope were infected (SM2). In contrast to our results, it was reported that *Ae. caspius* was infected with *D. repens* in Italy [38] and Moldova [58], with *D. immitis* in Serbia [37], Portugal [25] and Hungary, based on one positive sample of *D. repens* and *D. immitis* out of 267 collected mosquitoes from four species [71]. The absence of infection in *Ae. caspius* in our collections may be explained by there being no infection or only slight infection at that particular collection point, since specimens of other species were also negative.

In four species (*Ae. leucomelas*, *Ae. cataphylla, Ae. cinereus* and *Ae. excrucians*), *Dirofilaria* were found only in the abdomens, indicating that its development did not reach an infective L3 stage (Table 2). Also, it should be highlighted that in three species of mosquitoes (*Ae. cantans, Ae. communis,* and *Ae. vexans*), there were positive signals for *Dirofilaria* simultaneously in the thorax-head and abdomen pools. This fact may indicate that mosquitoes could ingest the filariae at different times and repeatedly, and not all nematodes managed to complete the development cycle to become infective larvae and migrate to the front of the body. Similarly, it cannot be excluded that not all filariae reach the infectious stage due to possible defense mechanisms activated by host cells, such as encapsulation, melanization, and coagulation [12, 21, 34]. However, in all mosquito species, except *Ae. aegypti* where only one pool was infected, the percentage of positive thorax-head pools was lower compared to abdomen pools.

According to published research, the development of *Dirofilaria* to the infective stage (L3) was recorded in Europe in the mosquito species *Ae. caspius, An. maculipennis, Ae. vexans, Ae. geniculatus, Ae. albopictus,* and *Cx. pipiens* (Table 4). On the basis of our results, eleven mosquito species are epidemiologically dangerous, when *Dirofilaria* undergo development to L3 (Table 2). Of particular interest are the species *Ae. geniculatus, Ae. communis, Ae. intrudens, Ae. cantans* and *Cx. modestus*, in which L3 were found more than once. *Ae. Aegypti, Ae. geniculatus*, *An. messeae* and *Ae. communis* have host effectiveness values ranging from 1.3 to 4.76. It should be noted that the efficacy of *Ae. aegypti* as a vector of *Dirofilaria* has been studied many times in laboratory conditions [34, 57, 62], where the microfilariae developed to third-stage larvae, but not in field-collected *Ae. aegypti.* In seven mosquito species, the host effectiveness was less than 1 (Table 2).

### *Wolbachia* and *Dirofilaria* infection in individual mosquitoes

Most mosquito species uninfected with *Wolbachia* showed higher epidemiological potential for *Dirofilaria* transmission in all studied regions (host effectiveness 0.41–4.76; EIR = 2.96–8.67, average EIR = 4.2). *Ae. cinereus* and *Cx. modestus* pools that had *Dirofilaria* were free from *Wolbachia* (Table 3).

*Dirofilaria* DNA was detected in abdomen pools of both *Wolbachia*-infected and uninfected mosquitoes. This result shows that *Wolbachia* does not prevent the acquisition of *Dirofilaria* by mosquitoes in nature. However, in eight thorax-head pools, *D. immitis* DNA was only detected in *Wolbachia*-uninfected mosquitoes. Moreover, after individual study of 11 thorax-heads from two *Wolbachia* positive *Cq. richiardii* abdomen pools, the *D. immitis* development was successful only in *Wolbachia*-free sample (Table SM4).

*D. repens* DNA in thorax-heads was found in 14 *Wolbachia*-uninfected and in only two *Wolbachia*-infected pools, in one of *Ae. albopictus* and in one of *Cx. pipiens*. We could not study mosquitoes from these two pools individually, so it is impossible to determine whether all the individuals in pool were infected with the bacterium, and to what ecological form (f. *pipiens* or f. *molestus*) of *Cx. pipiens* they belonged. Therefore, our findings do not prove a clear influence of bacteria on the development of *Dirofilaria*.

Nevertheless, the ratio of *Dirofilaria*-infective mosquitoes is much higher in *Wolbachia*-free mosquito specimens than in *Wolbachia*-infected, 22:2. Differences in the effects of different strains of *Wolbachia* were not recorded. However, given the small sample size of *Dirofilaria*-infected mosquitoes, further investigation into whether *Wolbachia* is present in individual *Cx. pipiens* and *Ae. albopictus* mosquitoes carrying infective L3 stage larvae is required.

It is known that artificial bacterial transfer significantly increases the expression of immune genes, including those involved in the Toll and IMD immune pathways, enhances the mosquito’s resistance to pathogens [5, 48, 49], and inhibits the development of filarial nematodes [1, 33]. In contrast, it has been shown that native *Wolbachia* does not affect the induction of host immune pathways [17, 39]. As a hypothesis, it could be proposed that there is a resource competition in the host for metabolites, because both *Dirofilaria* [19, 30] and *Wolbachia* [68] require them for their development. It should be noted that any *Wolbachia* anti-pathogen effect is dependent on bacterial density [40], so the development of microfilaria to the infective stage may differ in each mosquito. The study of simultaneous infection of individual mosquitoes with *Dirofilaria* spp. and a bacterial symbiont, taking into account *Wolbachia* density, will help us understand the mechanism of *Wolbachia* interference in the transmission of *Dirofilaria* by mosquitoes.

In conclusion, *Dirofilaria* were found in 15 mosquito species. This is the first study conducted in Russia examining the mosquito species as potential vectors of *D. immitis* and *D. repens*. Out of 1095 pools studied, there were 114 positive abdomen pools and 34 positive thorax-head pools. The ratio of infected pools to infective pools was 3.35:1. Mosquitoes in central temperate regions are able to spread *Dirofilaria* no less than mosquitoes in the southern regions. This indicates that the presence of infected dogs has a greater effect on the maintenance of foci of dirofilariasis than temperature. In the forests, the circulation of pathogens occurs with less intensity than in human settlements in rural and urban areas. For the first time in Europe, *Ae. aegypti, Ae. intrudens* and *Ae. communis* mosquitoes have been studied as *Dirofilaria* vectors, in which EIR values ranged from 3.08 to 5.33. Our data showed that *Ae. albopictus* and *Culex pipiens* s.l. are not the most important vectors of *Dirofilaria*. The greatest epidemiological danger was represented by *An. messeae, Ae. aegypti, Ae. geniculatus,* and *Ae. communis. Ae. cantans* might be added to this list given the considerable host effectiveness and the very high density.

## Supporting information

*SM1*. Mosquitoes collected between 2013 and 2017. Information on the specificities and the coordinates of the sampling site, sampling date, total mosquito number and *Dirofilaria* screening results. *SM2*. Mosquito infection with *Dirofilaria* spp. in specific sample sites. Information on the sampling date, mosquito species, pool size and *Dirofilaria* screening results. *SM3*. Occurrence of *Wolbachia* in mosquito species. Information on the sampling region, mosquito number, pool size and *Wolbachia* screening results. *SM4*. Comparison of simultaneous infection with *Wolbachia* and *Dirofilaria* individually. Supplementary materials are available at https://www.parasite-journal.org/10.1051/parasite/2019002/olm.

## References

[1] Andrews ES, Crain PR, Fu Y, Howe DK, Dobson SL. 2012 Reactive oxygen species production and *Brugia pahangi* survivorship in *Aedes polynesiensis* with artificial *Wolbachia* infection types. PLoS Pathogens, 8(12), e1003075. 23236284 10.1371/journal.ppat.1003075 PMC3516568

[2] Arakeljan R, Kovtunov A, Bikov V, Shatalin V, Arakeljan E. 2008 Epidemiologic-episootologic features of three-member system of dirofilariasis (dog-mosquito-people) on the territory of Astrakhan region. Siberian Medical Journal, 7, 13–18 (in Russian).

[3] Atyame CM, Delsuc F, Pasteur N, Weill M, Duron O. 2011 Diversification of *Wolbachia* endosymbiont in the *Culex pipiens* mosquito. Molecular Biology and Evolution, 28, 2761–2772. 21515811 10.1093/molbev/msr083

[4] Barashkova SV. 2011 Case of dirofilariasis in adolescent in Saint-Petersburg: Clinical and morphological characteristic. Journal Infectology, 3, 108–110 (in Russian).

[5] Bian G, Joshi D, Dong Y, Lu P, Zhou G, Pan X, Xu Y, Dimopoulos G, Xi Z. 2013 *Wolbachia* invades *Anopheles stephensis* populations and induces refractoriness to Plasmodium infection. Science, 340(6133), 748–751. 23661760 10.1126/science.1236192

[6] Biskin Z, Duziu O, Yildirim A, Inci A. 2010 The molecular diagnosis of *Dirofilaria immitis* in vector mosquitoes in Felahiye district of Kayseri. Turkiye Parazitoloji Dergisi, 34(3), 200–205. 20954124

[7] Bockova E, Rudolf I, Kocisova A, Betasova L, Venclikova K, Mendel J, Hubalek Z. 2013 *Dirofilaria repens* microfilariae in *Aedes vexans* mosquitoes in Slovakia. Parasitology Research, 112(10), 3465–3470. 23846240 10.1007/s00436-013-3526-9 PMC3779099

[8] Braig HR, Zhou W, Dobson SL, O’Neill SL. 1998 Cloning and characterization of a gene encoding the major surface protein of the bacterial endosymbiont *Wolbachia pipientis*. Journal of Bacteriology, 180(9), 2373–2378. 9573188 10.1128/jb.180.9.2373-2378.1998 PMC107178

[9] Bravo-Barriga D, Parreira R, Almeida APG, Calado M, Blanco-Ciudad J, Serrano-Aguilera FJ, Perez-Martin JE, Sanchez-Peinado J, Pinto J, Reina D, Frontera E. 2016 *Culex pipiens* as a potential vector for transmission of *Dirofilaria immitis* and other unclassified Filarioidea in Southwest Spain. Veterinary Parasitology, 223, 173–180. 27198797 10.1016/j.vetpar.2016.04.030

[10] Byakova OV, Maslennikova OV, Ermolina SA. 2014 Dirofilariosis dog in the Kirov region. Basic Research, 11, 1297–1300 (in Russian).

[11] Cabanova V, Miterpakova M, Valentova D, Blazejova H, Rudolf I, Stloukal E, Hurnikova Z, Dzidova M. 2018 Urbanization impact on mosquito community and the transmission potential of filarial infection in Central Europe. Parasites & Vectors, 11(1), 261. 29690912 10.1186/s13071-018-2845-1 PMC5937826

[12] Cancrini G, Frangipane di Regalbono A, Ricci I, Tessarin C, Gabrielli S, Pietrobelli M. 2003a *Aedes albopictus* is a natural vector of *Dirofilaria immitis* in Italy. Veterinary Parasitology, 118(3–4), 195–202. 14729167 10.1016/j.vetpar.2003.10.011

[13] Cancrini G, Romi R, Gabrielli S, Toma L, Di Paolo M, Scaramozzino P. 2003b First finding of *Dirofilaria repens* in a natural population of *Aedes albopictus*. Medical and Veterinary Entomology, 17(4), 448–451. 14651660 10.1111/j.1365-2915.2003.00463.x

[14] Cancrini G, Magi M, Gabrielli S, Arispici M, Tolari F, Dell’Omodarme M, Prati MC. 2006 Natural vectors of dirofilariasis in rural and urban areas of the Tuscan region, central Italy. Journal of Medical Entomology, 43(3), 574–579. 16739418 10.1603/0022-2585(2006)43[574:nvodir]2.0.co;2

[15] Cancrini G, Gabrielli S. 2007 Vectors of Dirofilaria nematodes: biology, behaviour and host/parasite relationships, in *Dirofilaria immitis* and *D. repens* in dog and cat and human infections, Genchi C, Rinaldi L, Cringoli G, Editors. Veterinary Parasitology and Parasitic Diseases, Department of Pathology and Animal Health, Faculty of Veterinary Medicine, University of Naples Federico II: Napoli, NA, Italy p. 48–58. ISBN 9788889132142.

[16] Cancrini G, Scaramozzino P, Gabrielli S, Di Paolo M, Toma L, Romi R. 2007 *Aedes albopictus* and *Culex pipens* implicated as natural vectors of *Dirofilaria repens* in central Italy. Journal of Medical Entomology, 44(6), 1064–1066. 18047207 10.1603/0022-2585(2007)44[1064:aaacpi]2.0.co;2

[17] Caragata EP, Pais FS, Baton LA, Silva JBL, Sorgine MHF, Moreira LA. 2017 The transcriptome of the mosquito *Aedes fluviatilis* (Diptera: Culicidae), and transcriptional changes associated with its native *Wolbachia* infection. BMC Genomics, 18, 6. 28049478 10.1186/s12864-016-3441-4 PMC5210266

[18] Castillo JC, Reynolds SE, Eleftherianos I. 2011 Insect immune responses to nematode parasites. Trends in Parasitology, 27(12), 537–547. 21982477 10.1016/j.pt.2011.09.001

[19] Cotton JA, Bennuru S, Grote A, Harsha B, Tracey A, Beech R, Dovie SR, Dunn M, Hotopp JC, Holroyd N, Kikuchi T, Lambert O, Mhashilkar A, Mutowo P, Nursimulu N, Ribeiro JM, Rogers MB, Stanley E, Swapna LS, Tsai IJ, Unnasch TR, Voronin D, Parkinson J, Nutman TB, Ghedin E, Berriman M, Lustigman S. 2016 The genome of *Onchocerca volvulus*, agent of river blindness. Nature Microbiology, 2, 16216. 10.1038/nmicrobiol.2016.216 PMC5310847 27869790

[20] Cowling DW, Gardner IA, Johnson WO. 1999 Comparison of methods for estimation of individual-level prevalence based on pooled samples. Preventive Veterinary Medicine, 39(3), 211–225. 10327439 10.1016/s0167-5877(98)00131-7

[21] De Carvalho GA, Ramos RAN, Trindade Maia R, de Andrade CFS, Alves CL. 2018 Melanization of *Dirofilaria immitis* larvae in different culicid species. Journal of Arthropod-Borne Diseases, 12(1), 94–99. 30018997 PMC6046106

[22] de Pinho Mixao V, Mendes AM, Mauricio IL, Calado MM, Novo MT, Bello S, Almeida AP. 2016 Molecular detection of *Wolbachia pipientis* in natural populations of mosquito vectors of *Dirofilaria immitis* from continental Portugal: first detection in *Culex theileri*. Medical and Veterinary Entomology, 30, 301–309. 27279553 10.1111/mve.12179

[23] Dyab AK, Galal LA, Mahmoud AE, Mokhtar Y. 2016 Finding *Wolbachia* in Filarial larvae and Culicidae mosquitoes in Upper Egypt governorate. Korean Journal of Parasitology, 54(3), 265–272. 27417080 10.3347/kjp.2016.54.3.265 PMC4977788

[24] Ermakova L, Nagorny S, Krivorotova E, Pshenichnaya N, Matina O. 2014 *Dirofilaria repens* in the Russian Federation: current epidemiology, diagnosis, and treatment from a federal reference center perspective. International Journal of Infectious Diseases, 23, 47–52. 24661932 10.1016/j.ijid.2014.02.008

[25] Ferreira CA, de Pinho MV, Novo MT, Calado MM, Gonçalves LA, Belo SM, de Almeida AP. 2015 First molecular identification of mosquito vectors of *Dirofilaria immitis* in continental Portugal. Parasites & Vectors, 8, 139 DOI: 10.1186/s13071-015-0760-2. 25886610 PMC4369802

[26] Ganushkina LA, Rakova VM, Ivanova IB, Supriaga VG, Sergiev VP. 2014 Entomological monitoring of an area to assess *Dirofilaria* transmission risk. Meditsinskaia Parazitologiia i Parazitarnye Bolezni (Mosk), 3, 9–12. 25286542

[27] Genchi C, Rinaldi L, Mortarino M, Genchi M, Cringoli G. 2009 Climate and *Dirofilaria infection* in Europe. Veterinary Parasitology, 163(4), 286–292. 19398159 10.1016/j.vetpar.2009.03.026

[28] Giangaspero A, Marangi M, Latrofa MS, Martinelli D, Traversa D, Otranto D, Genchi C. 2013 Evidences of increasing risk of dirofilarioses in southern Italy. Parasitology Research, 112(3), 1357–1361. 23224639 10.1007/s00436-012-3206-1

[29] Gouveia M. 2007 Susceptibility of Mosquito Vectors to *Dirofilaria immitis* on Madeira Island, Portugal. Tese Doutoramenteo Universidade da Madeira Funchal, Portugal: Universidade da Madeira 113 p. hdl.handle.net/10400.13/27.

[30] Grote A, Voronin D, Ding T, Twaddle A, Unnasch TR, Lustigman S, Ghedin E. 2017 Defining *Brugia malayi* and *Wolbachia* symbiosis by stage-specific dual RNA-seq. PLoS Neglected Tropical Diseases, 11(3), e0005357. 28358880 10.1371/journal.pntd.0005357 PMC5373514

[31] Gutsevich AV, Monchadskii AS, Shtakelberg AA. 1970 Fauna of the USSR. Diptera. Mosquitoes. Nauka: Leningrad (in Russian).

[32] Hertig M, Wolbach SB. 1924 Studies on rickettsia-like micro-organisms in insects. Journal of Medical Research, 44(3), 329–374. 19972605 PMC2041761

[33] Kambris Z, Cook PE, Phuc HK, Sinkins SP. 2009 Immune activation by life-shortening *Wolbachia* and reduced filarial competence in mosquitoes. Science (New York, NY), 326(5949), 134–136. 10.1126/science.1177531 PMC2867033 19797660

[34] Kartman L. 1953 Factors influencing infection of the mosquito with *Dirofilaria immitis* (Leidy, 1856). Experimental Parasitology, 2(1), 27–78.

[35] Krivorotova EY. 2016 Xenomonitoring of dirofilariasis in the south and north-west of the Russian Federation. Parazitologiia, 50(5), 357–364 (in Russian). 29211425

[36] Kronefeld M, Kampen H, Sassnau R, Werner D. 2014 Molecular detection of *Dirofilaria immitis, Dirofilaria repens* and *Setaria tundra* in mosquitoes from Germany. Parasites & Vectors, 7(1), 30. 24433279 10.1186/1756-3305-7-30 PMC3898823

[37] Kurucz K, Kepner A, Krtinic B, Zana B, Foldes F, Földes F, Bányai K, Oldal M, Jakab F, Kemenesi G. 2016 First molecular identification of *Dirofilaria spp*. (Onchocercidae) in mosquitoes from Serbia. Parasitology Research, 115(8), 3257–3260. 27193348 10.1007/s00436-016-5126-y

[38] Latrofa MS, Dantas-Torres F, Annoscia G, Genchi M, Traversa D, Otranto D. 2012 A duplex real-time polymerase chain reaction assay for the detection of and differentiation between *Dirofilaria immitis* and *Dirofilaria repens* in dogs and mosquitoes. Veterinary Parasitology, 185(2–4), 181–185. 22119343 10.1016/j.vetpar.2011.10.038

[39] Li J, Wang N, Liu Y, Qiu S. 2018 Proteomics of *Nasonia vitripennis* and the effects of native *Wolbachia* infection on *N. vitripennis*. PeerJ, 6, e4905. 29868291 10.7717/peerj.4905 PMC5978391

[40] Lu P, Bian G, Pan X, Xi Z. 2012 *Wolbachia* induces density-dependent inhibition to dengue virus in mosquito cells. PLoS Neglected Tropical Diseases, 6(7), e1754. 22848774 10.1371/journal.pntd.0001754 PMC3404113

[41] Masetti A, Rivasi F, Bellini R. 2008 Mosquito-based survey for the detection of flaviviruses and filarial nematodes in *Aedes albopictus* and other anthropophilic mosquitoes collected in northern Italy. New Microbiolica, 31(4), 457–465. 19123300

[42] Mckay T, Bianco T, Rhodes L, Barnett S. 2013 Prevalence of *Dirofilaria immitis* (Nematoda: Filarioidea) in mosquitoes From Northeast Arkansas, the United States. Journal of Medical Entomology, 50(4), 871–878. 23926787 10.1603/me12197

[43] Montarsi F, Ciocchetta S, Devine G, Ravagnan S, Mutinelli F, Frangipane di Regalbono A, Otranto D, Capelli G. 2015 Development of *Dirofilaria immitis* within the mosquito *Aedes* (*Finlaya*) *koreicus*, a new invasive species for Europe. Parasites & Vectors, 8(1), 177. 25884876 10.1186/s13071-015-0800-y PMC4382832

[44] Moodley K, Govin CN, Peer AKC, Westhuizen MVD, Parbhoo D, Ming Sun L, du Plessis DC, Frean JA. 2015 First detection of human dirofilariasis in south Africa. Infectious Disease Reports, 7(1), 5726. 25874068 10.4081/idr.2015.5726 PMC4387369

[45] Morchón R, Bargues MD, Latorre JM, Melero-Alcíbar R, Pou-Barreto C, Mas-Coma S, Simon F. 2007 Haplotype H1 of *Culex pipiens* implicated as a natural vector of *Dirofilaria immitis* in an endemic area of western Spain. Vector Borne and Zoonotic Disease, 7(4), 653–658. 10.1089/vbz.2007.0124 17979532

[46] Murata K, Yanai T, Agatsuma T, Uni S. 2003 *Dirofilaria immitis* Infection of a Snow Leopard (*Uncia uncia*) in a Japanese Zoo with mitochondrial DNA analysis. Journal of Veterinary Medical Science, 65(8), 945–947. 12951432 10.1292/jvms.65.945

[47] Nicolescu G, Linton YM, Vladimirescu A, Howard TM, Harbach RE. 2004 Mosquitoes of the *Anopheles maculipennis* group (Diptera: Culicidae) in Romania, with the discovery and formal recognition of new species based on molecular and morphological evidence. Bulletin of Entomological Research, 94(6), 525–535. 15541192 10.1079/ber2004330

[48] Pan X, Zhou G, Bian G, Lu P, Raikhel AS, Xi Z. 2012 *Wolbachia* induces reactive oxygen species (ROS)-dependent activation of the Toll pathway to control dengue virus in the mosquito *Aedes aegypti*. Proceedings of the National Academy of Sciences, 109(1), E23–E31. 10.1073/pnas.1116932108 PMC3252928 22123956

[49] Pan X, Pike A, Joshi D, Bian G, McFadden MJ, Lu P, Liang X, Zhang F, Raikhel AS, Xi Z. 2018 The bacterium *Wolbachia* exploits host innate immunity to establish a symbiotic relationship with the dengue vector mosquito *Aedes aegypti*. ISME Journal, 12(1), 277–288. 29099491 10.1038/ismej.2017.174 PMC5739022

[50] Paras KL, O’Brien VA, Reiskind MH. 2014 Comparison of the vector potential of different mosquito species for the transmission of heartworm, *Dirofilaria immitis*, in rural and urban areas in and surrounding Stillwater, Oklahoma, U.S.A. Medical and Veterinary Entomology, 28(Suppl 1), 60–67. 10.1111/mve.12069 24898348

[51] Ricci I, Cancrini G, Gabrielli S, Damelio S, Favia G. 2002 Searching for *Wolbachia* (Rickettsiales: Rickettsiaceae) in mosquitoes (Diptera: Culicidae): Large polymerase chain reaction survey and new identifications. Journal of Medical Entomology, 39, 562–567. 12144285 10.1603/0022-2585-39.4.562

[52] Rudolf I, Šebesta O, Mendel J, Betášová L, Bocková E, Jedličková P, Venclíková K, Blažejová H, Šikutová S, Hubálek Z. 2014 Zoonotic *Dirofilaria repens* (Nematoda: Filarioidea) in *Aedes vexans* mosquitoes, Czech Republic. Parasitology Research, 113, 4663–4667. 25346197 10.1007/s00436-014-4191-3

[53] Ryabova T, Yunicheva Y, Markovich N, Ganushkina L, Orabei V, Sergiev V. 2005 Detection of *Aedes (Stegomyia) aegypti* L. mosquitoes in Sochi. Meditsinskaia parazitologiia i parazitarnye bolezni, 3, 3–5 (in Russian). 16212085

[54] Sergiev VP, Supriaga VG, Bronshteĭn AM, Ganushkina LA, Rakova VM, Morozov EN, Fedianina LV, Frolova AA, Morozova LF, Ivanova IB, Darchenkova NN, Zhukova LA. 2014 Results of studies of human dirofilariasis in Russia. Meditsinskaia parazitologiia i parazitarnye bolezni, 3, 3–9 (in Russian). 25286541

[55] Shaikevich E. 2007 PCR-RFLP of the COI gene reliably differentiates *Cx. pipiens*, *Cx. pipiens* f. *molestus* and *Cx. torrentium* of the Pipiens Complex. European Mosquito Bulletin, 23, 25–30.

[56] Shaikevich E, Vinogradova E, Bouattour A, Almeida APG. 2016 Genetic diversity of *Culex pipiens* mosquitoes in distinct populations from Europe. Contribution of *Cx. quinquefasciatus* in Mediterranean populations. Parasites & Vectors, 9(1), 47. 26818097 10.1186/s13071-016-1333-8 PMC4730663

[57] Sulaiman I, Towson H. 1980 The genetic basis of susceptibility of infection with *Dirofilaria immitis* in *Aedes aegypti*. Annals of Tropical Medicine and Parasitology, 74, 635–646. 7458468 10.1080/00034983.1980.11687397

[58] Sulesco T, von Thien H, Toderas L, Toderas I, Lühken R, Tannich E. 2016 Circulation of *Dirofilaria immitis* in Moldova. Parasites & Vectors, 9(1), 627. 27912786 10.1186/s13071-016-1916-4 PMC5135815

[59] Sulesco T, von Thien H, Toderas L, Toderas I, Lühken R, Tannich E. 2016 Detection of Dirofilaria repens and Dirofilaria immitis DNA in mosquitoes from Belarus. Parasitology Research, 115, 3535–3541. 27169723 10.1007/s00436-016-5118-y

[60] Tarello W. 2011 Clinical aspects of dermatitis associated with *Dirofilaria repens* in pets: a review of 100 canine and 31 feline cases (1990–2010) and a report of a new clinic case imported from Italy to Dubai. Journal of Parasitology Research. 10.1155/2011/578385 PMC3238394 22203888

[61] Taylor MJ, Voronin D, Johnston KL, Ford L. 2013 *Wolbachia* filarial interactions. Cellular Microbiology, 15(4), 520–526. 23210448 10.1111/cmi.12084

[62] Tiawsirisup S, Nithiuthai S. 2006 Vector competence of *Aedes aegypti* (L.) and *Culex quinquefasciatus* (Say) for *Dirofilaria immitis* (Leidy). Southeast Asian Journal of Tropical Medicine and Public Health, 37(suppl 3), 110–114. 17547063

[63] Tumolskaya NI, Pozio E, Rakova VM, Supriaga VG, Sergiev VP, Morozov EN, Morozova LF, Rezza G, Litvinov SK. 2016 *Dirofilaria immitis* in a child from the Russia Federation. Parasite, 23, 37. 27600944 10.1051/parasite/2016037 PMC5018928

[64] Vakalis N, Spanakos G, Patsoula E, Vamvakopoulos NC. 1999 Improved detection of *Dirofilaria repens* DNA by direct polymerase chain reaction. Parasitology International, 48, 145–150. 11269275 10.1016/s1383-5769(99)00012-4

[65] Werren JH, Baldo L, Clark ME. 2008 *Wolbachia*: master manipulators of invertebrate biology. Nature Reviews. Microbiology, 6, 741–751. 18794912 10.1038/nrmicro1969

[66] Wilkerson RC, Linton Y-M, Fonseca DM, Schultz TR, Price DC, Strickman DA. 2015 Making mosquito taxonomy useful: a stable classification of Tribe Aedini that balances utility with current knowledge of evolutionary relationships. PLoS One, 10(7), e0133602. 26226613 10.1371/journal.pone.0133602 PMC4520491

[67] Wright JD, Barr AR. 1980 The ultrastructure and symbiotic relationships of *Wolbachia* of mosquitoes of the *Aedes scutellaris* group. Journal of Ultrastructure Research, 72, 52–64. 7411685 10.1016/s0022-5320(80)90135-5

[68] Wu M, Sun LV, Vamathevan J, Riegler M, Deboy R, Brownlie JC, McGraw EA, Martin W, Esser C, Ahmadinejad N, Wiegand C, Madupu R, Beanan MJ, Brinkac LM, Daugherty SC, Durkin AS, Kolonay JF, Nelson WC, Mohamoud Y, Lee P, Berry K, Young MB, Utterback T, Weidman J, Nierman WC, Paulsen IT, Nelson KE, Tettelin H, O’Neill SL, Eisen JA. 2004 Phylogenomics of the 235 reproductive parasite *Wolbachia pipientis* wMel: a streamlined genome overrun by mobile genetic elements. PLoS Biology, 2(3), e69. 15024419 10.1371/journal.pbio.0020069 PMC368164

[69] Yildirim A, Inci A, Duzlu O, Biskin Z, Ica A, Sahin I. 2011 *Aedes vexans* and *Culex pipiens* as the potential vectors of *Dirofilaria immitis* in Central Turkey. Veterinary Parasitology, 178(1–2), 143–147. 21232866 10.1016/j.vetpar.2010.12.023

[70] Zhou W, Rousset F, O’Neil S. 1998 Phylogeny and PCR-based classification of *Wolbachia* strains using *wsp* gene sequences. Proceedings of the Royal Society B: Biological Sciences, 265(1395), 509–515. 10.1098/rspb.1998.0324 PMC1688917 9569669

[71] Zittra C, Kocziha Z, Pinnyei S, Harl J, Kieser K, Laciny A, Eigner B, Silbermayr K, Duscher GG, Fok E, Fuehrer HP. 2015 Screening blood-fed mosquitoes for the diagnosis of filarioid helminthes and avian malaria. Parasistes & Vectors, 8, 16. 10.1186/s13071-015-0637-4 PMC4305256 25582219

